# Retrospective analysis of mortality among children under 5 years of age in Huangshi over the period 2002–2022, China

**DOI:** 10.1186/s12889-024-18955-3

**Published:** 2024-05-29

**Authors:** Jumin Xie, Yihan Hong, Jianlin Yang, Yueming Yan, Shinuan Fei

**Affiliations:** 1 Hubei Key Laboratory of Renal Disease Occurrence and Intervention, School of Medicine, Hubei Polytechnic University, Guilin north road No 16, Huangshi, Hubei 435003 P. R. China; 2 Pediatrics Department, Huangshi Maternal and Child Health Care Hospital, Guilin south road No 9, Huangshi, Hubei 435003 P. R. China

**Keywords:** Causes of death, Children, Mortality, U5MR

## Abstract

**Background:**

The United Nations’ Millennium Development Goals and Sustainable Development Goals both underscore the critical need to reduce the under-five mortality rate globally. China has made remarkable progress in decreasing the mortality rate of children under five. This study aims to examine the trends in child mortality rates from 2002 to 2022 and the causes of deaths among neonates, infants, and children under 5 years of age from 2013 to 2022 in Huangshi.

**Methods:**

The data resource was supported and provided by the Huangshi Health Commission, Huangshi Maternal and Child Health Hospital, and the Huangshi Statistics Bureau. Figures were drawn using Origin 2021.

**Results:**

The mortality rate among children under 5 years old significantly decreased, from 21.38 per 1,000 live births in 2002 to 3.53 per 1,000 live births in 2022. The infant mortality rate also saw a significant decline, to 15.06 per 1,000 live births. Among the 1,929 recorded child deaths from 2013 to 2022, the top three causes were: F2 (Disorders related to short gestation and low birth weight), accounting for 17.26% (333 deaths); I1 (Accidental drowning and submersion), for 14.83% (286 deaths); and I3 (Other accidental threats to breathing), for 12.29% (237 deaths). Of the 1,929 deaths, 1,117 were male children, representing 57.91%. The gender disparity in the Under-5 Mortality Rate (U5MR) was calculated to be 1.38 (boys to girls). The leading causes of death under the age of five shifted from F2 (Disorders related to short gestation and low birth weight) to I1 (Accidental drowning and submersion) as children aged, highlighting the need for policymakers and parents to intensify care and vigilance for children.

**Conclusions:**

Huangshi has achieved significant progress in lowering child mortality rates over the past two decades. The study calls for policymakers to enact more effective measures to further reduce the mortality rate among children under 5 years of age in Huangshi. Furthermore, it advises parents to dedicate more time and effort to supervising and nurturing their children, promoting a safer and healthier development.

**Supplementary Information:**

The online version contains supplementary material available at 10.1186/s12889-024-18955-3.

## Introduction

In September 2000, the 189 member states of the United Nations collectively initiated the Millennium Development Goals (MDGs) to address pivotal global challenges including poverty, hunger, disease, illiteracy, environmental degradation, and gender inequality [1]. The fourth MDG specifically aimed to achieve a two-thirds reduction in the under-five mortality rate (U5MR) by 2015, using 1990 levels as a benchmark [2]. Despite concerted efforts, about 5.4 million children under five years of age died in 2017, with nearly 2.5 million of these deaths occurring in the first month of life [3]. The MDGs were then succeeded by the Sustainable Development Goals (SDGs), introduced by the United Nations General Assembly in 2015. One of the SDGs’ targets is to lower the U5MR to at most 25 per 1,000 live births by 2030 [4]. Notably, there has been significant progress, with a 59% reduction in U5MR from 93.0 per 1,000 live births in 1990 to 37.7 in 2019 [4]. Goal 3 of the SDGs focuses on ending preventable deaths among newborns and children under five by 2030 [5].

China has made significant progress in reducing child mortality, with the U5MR declining by two-thirds from 1990 to 2015 [6]. Furthermore, China has achieved several health-related objectives set forth in the Sustainable Development Agenda ahead of the 2030 deadline. The U5MR was reduced to no more than 25 per 1,000 live births by 2004, and the neonatal mortality rate (NMR) to no more than 12 per 1,000 live births by 2006 [7]. Remarkably, the U5MR decreased from 61 per 1,000 live births in 1991 to 7.1 per 1,000 live births in 2021, highlighting the Chinese government’s success in managing child mortality rates effectively [8]. From 2002 to 2021, the global U5MR decreased from 71.2 to 38.1 per 1,000 live births, while China’s U5MR fell from 31.5 to 6.9 per 1,000 live births (WHO data). However, significant disparities in U5MR and NMR persist across different ethnic groups, and between urban and rural areas, as well as developed and underdeveloped regions [7].

Huangshi, a prefecture-level city in Hubei Province and an integral part of the Wuhan Metropolitan Area, is located in the southeastern part of the province. As of 2022, Huangshi Municipality’s administrative division includes four districts, one county, and one county-level city. By the end of 2022, the city’s permanent population had reached 2.444 million, with an urban population of 1.665 million, constituting 68.13% of the total. In 2022, Huangshi’s GDP reached 204.151 billion yuan, registering a growth of 5.6%. By the end of 2023, the per capita disposable income of urban residents had reached 46,794 yuan, reflecting a 5.4% increase from the previous year; meanwhile, the per capita disposable income of rural residents was 21,757 yuan, showing an 8.1% year-on-year increase.

With the rapid advancement of China’s healthcare sector, the mortality rate of children under the age of five has steadily declined. This study gathered data on U5MR from 2002 to 2022 in Huangshi and analyzed the trend of mortality among children under 5 years old over the past 20 years. It examined the primary causes of death for neonate, infants, and children under 5, as well as their trends and sex ratios from 2013 to 2022, with the aim of proposing effective interventions to further reduce under-5 mortality.

Moreover, this paper highlights the crucial need for parents to devote more time and effort to nurturing and caring for their children. It emphasizes the importance of creating a healthier and more supportive environment for children’s growth. This not only serves as a reference for policymakers but also reminds parents to contribute to creating an optimal developmental setting for their children.

## Materials and methods

### Definitions

The under-five mortality rate (U5MR), as defined by the World Health Organization (WHO) and outlined in Sustainable Development Goal (SDG) 3.2.1, represents the probability (expressed per 1,000 live births) that a newborn in a given year or period will die before reaching the age of five, assuming current age-specific mortality rates persist [9]. This definition aligns with WHO standards for calculating the U5MR [10, 11].

The definition of live births adheres to guidelines set by the United Nations Statistics Division [12]. Furthermore, according to *The Health Industry Standards of the People’s Republic of China*, specifically indicators of health statistics, Part 5: Maternal and child health care, live births are defined as infants born with a gestational age of at least 28 weeks. In instances where gestational age is uncertain, a birth weight of 1,000 g or more serves as a criterion.

### Data sources

Data on mortality rates for children under the age of five in Huangshi, covering the years 2002 to 2022, were collected by the Huangshi Maternal and Child Health Hospital. The Huangshi Health Commission approved the collection process, and data were compiled from all hospitals and regional health commissions within Huangshi. Information on Huangshi’s total population, number of births, and birth rate were exclusively obtained from the Huangshi Statistical Bureau.

### Inclusion and exclusion criteria

In this study, a total of 4,342 deaths among children under the age of five were recorded from 2002 to 2022 in Huangshi.

The research included a cohort of 1,929 children under five, who had complete case records and met the diagnostic criteria, during the period from 2013 to 2022. Furthermore, 61 children with incomplete data were excluded from the analysis.

The mortality data for children under five in Huangshi from 2002 to 2012, which consisted only of statistical summaries without recorded causes of death, was not included in the analysis of causes of death for this age group.

### Causes of child death

The causes of death in children under the age of five were systematically classified according to the ICD-10 (10th edition) International Classification of Diseases. The terminology and classification of these causes were established by consulting the “*Diagnosis, Reporting, and Classification of Causes of Death in Children under 5 Years of Age*” guidelines issued by the China Maternal and Child Health Monitoring System [13], which aligns with the ICD-10 framework for cause of death classification. Mortality etiologies were divided into 35 distinct subcategories, as detailed in Supplemental Table 1, also based on the ICD-10 standards.

## Results

### Birth records and rate in Huangshi from 2002 to 2022

**Fig. 1:**
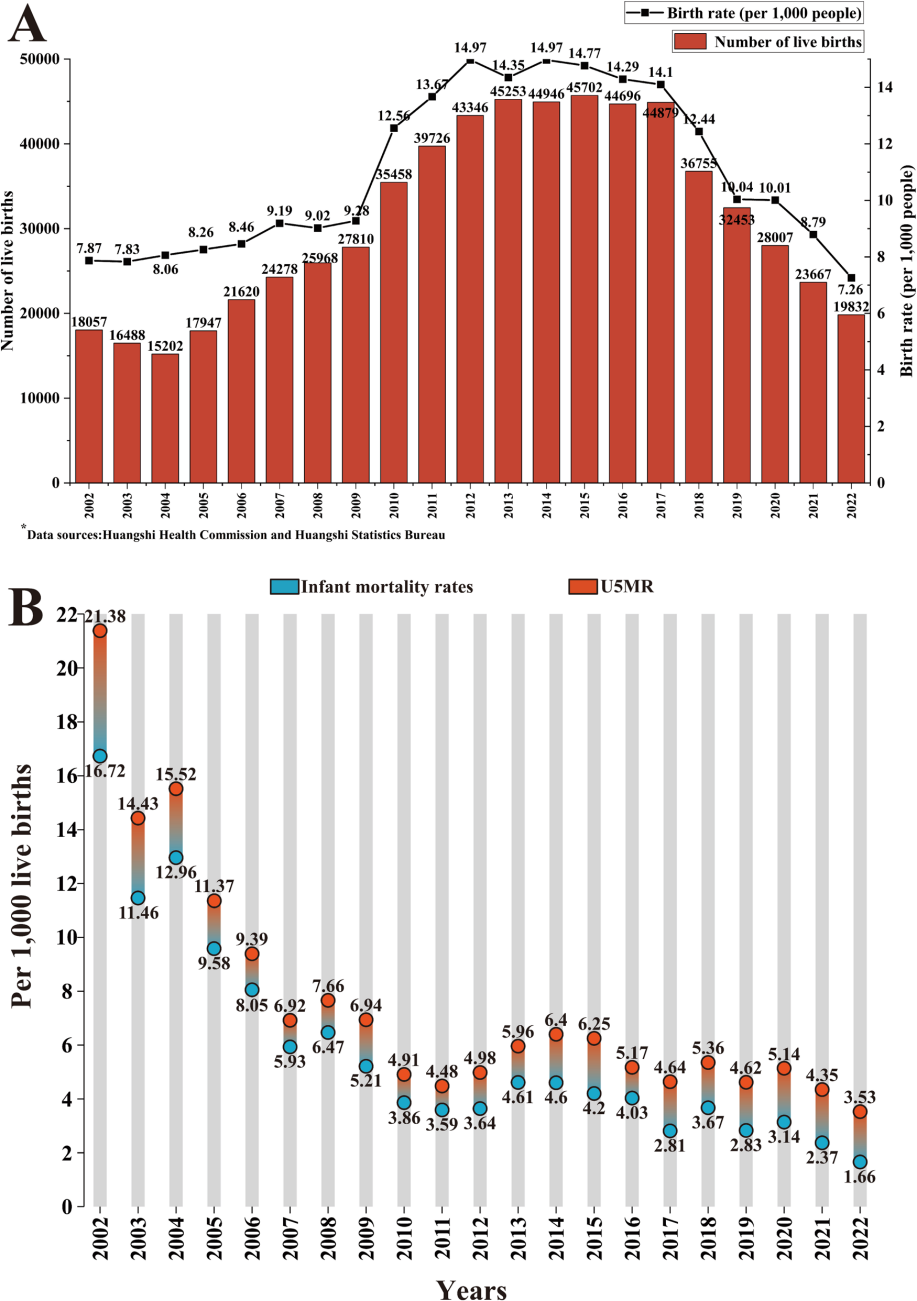
Trends in live births, birth rate, and under-5 mortality rate in Huangshi, 2002–2022. **A** Changes in the number of live births and birth rate in Huangshi, 2002–2022. **B** Trends in under-5 mortality rate and infant mortality rate in Huangshi, 2002–2022. The data were provided by the Huangshi Health Commission and Huangshi Statistics Bureau

The data on births and birth rates in Huangshi from 2002 to 2022 are summarized in (Fig. 1A). Notably, there was a steady yearly count of over 30,000 newborns from 2010 to 2019, peaking at more than 40,000 annually between 2012 and 2017, with the highest number recorded in 2015. The total live births amounted to 366,190 from 2013 to 2022, and 285,900 from 2002 to 2012. The birth rate paralleled the number of births, showing a significant decline since 2017. The lowest point was reached in 2022, with a birth rate of 7.26 per 1,000 live births, marking the lowest rate since the early 21st century.

### Infant mortality rate and U5MR in Huangshi from 2002 to 2022

Over the last two decades, infant and under-five child mortality rates have significantly decreased. Infant mortality rates dropped from 16.72 to 1.66 per 1,000 live births, and U5MR decreased from 21.38 to 3.53 per 1,000 live births, as shown in (Fig. 1B).

For a detailed comparison of the U5MR trends, we consulted precise data from the WHO. In 2021, Huangshi’s U5MR was 4.4 per 1,000 live births, which has been consistently lower than both China’s and the global rates over the past two decades, as depicted in (Supplemental Fig. 1).

A key insight from Fig. 1 is the divergent trends between the number of live births and under-five deaths.

### Major causes of death among children under five years of age

**Fig. 2:**
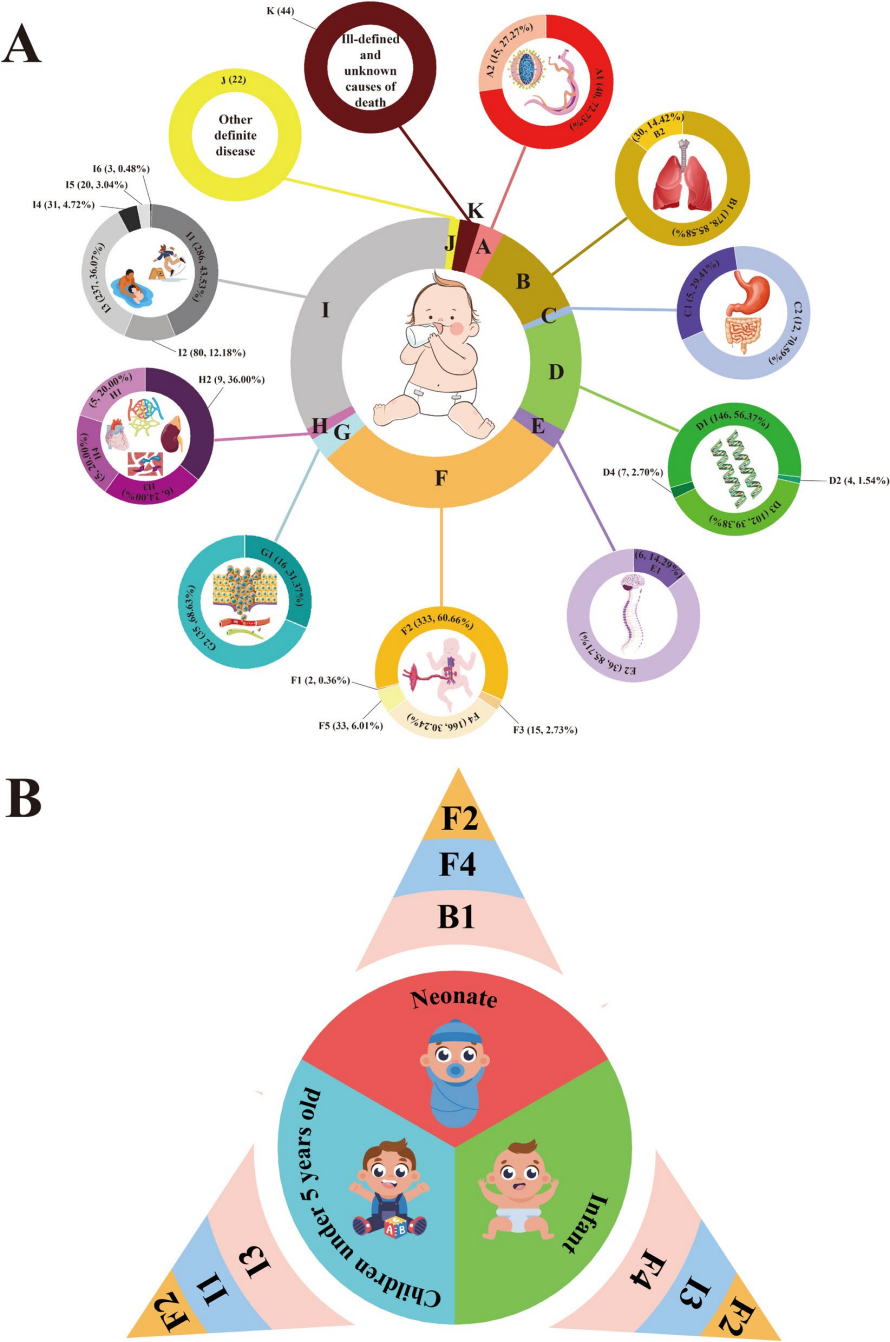
Causal classification of child mortality. **A** Distribution of causes of child deaths in Huangshi, 2013–2022. **B** Top 3 causes of mortality in neonates, infants, and children under 5 years of age

Between 2013 and 2022, a total of 1,929 child fatalities were recorded in Huangshi, as shown in (Fig. 2A). These deaths were classified into 11 primary causes (labeled A to K), further divided into 35 subcategories, detailed in (Supplemental Table 1).

The predominant causes of mortality among children under 5 years included: F2: Disorders related to short gestation and low birth weight, accounting for 333 deaths (17.26%); I1: Accidental drowning and submersion, with 286 deaths (14.83%); and I3: Other accidental threats to breathing, resulting in 237 deaths (12.29%), as illustrated in (Fig. 2B).

Regarding neonatal mortality, the leading factors were: F2: Disorders related to short gestation and low birth weight, with 303 deaths (35.86%); F4: Birth asphyxia, accounting for 161 deaths (19.05%); and B1: Pneumonia, resulting in 74 deaths (8.76%), as indicated in (Fig. 2B).

For infant mortality, the three main causes were: F2: Disorders related to short gestation and low birth weight, with 331 deaths (25.66%); I3: Other accidental threats to breathing, contributing to 186 deaths (14.42%); and F4: Birth asphyxia, resulting in 165 deaths (12.79%), as depicted in (Fig. 2B).

### Gender disparity in under-5 mortality rates

**Fig. 3:**
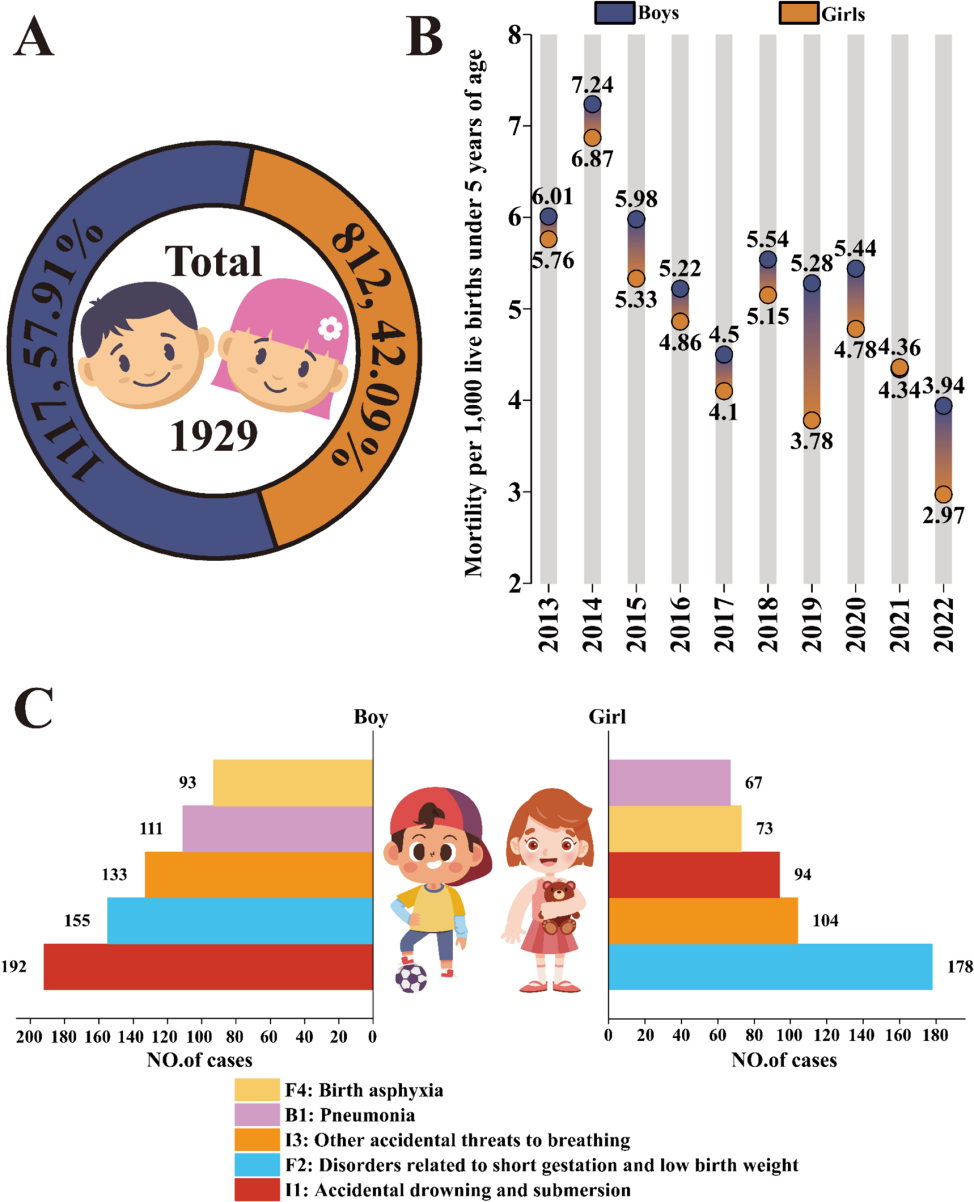
Gender disparities in child mortality. **A** Under-5 mortality rate (U5MR) disparities between boys and girls in Huangshi, 2013–2022. **B** Distribution of deaths by gender. **C** Leading causes of death in boys and girls under the age of 5: a ranking of the top 5 causes

The U5MR by gender in Huangshi from 2013 to 2022 revealed that out of the total 1,929 child deaths, 1,117 were boys (57.91%), while 812 were girls (42.09%), as shown in (Fig. 3A). This indicates a gender disparity ratio of 1.38.

Throughout the decade, the mortality rate fluctuated but showed a general decline, with boys consistently experiencing a higher mortality rate than girls each year, as demonstrated in (Fig. 3B).

The primary causes of mortality among boys were identified as: I1: Accidental drowning and submersion (192 deaths, 17.19%), F2: Disorders related to short gestation and low birth weight (155 deaths, 13.88%), I3: Other accidental threats to breathing (133 deaths, 11.91%), B1: Pneumonia (111 deaths, 9.94%), and F4: Birth asphyxia (93 deaths, 8.33%). In contrast, the leading causes of death among girls included: F2: Disorders related to short gestation and low birth weight (178 deaths, 21.92%), I3: Other accidental threats to breathing (104 deaths, 12.81%), I1: Accidental drowning and submersion (94 deaths, 11.58%), F4: Birth asphyxia (73 deaths, 8.99%), and B1: Pneumonia (67 deaths, 8.25%), as shown in (Fig. 3C).

Although the top five causes of death were consistent for both boys and girls, the order differed. A greater proportion of boys (325, 29.1%) died from accidents than girls (198, 24.39%), suggesting boys are more prone to engaging in risky, adventurous behavior compared to girls, as depicted in (Fig. 3C).

### The changes in the leading causes of death among children under 5 years of age in Huangshi from 2013 to 2022

**Fig. 4:**
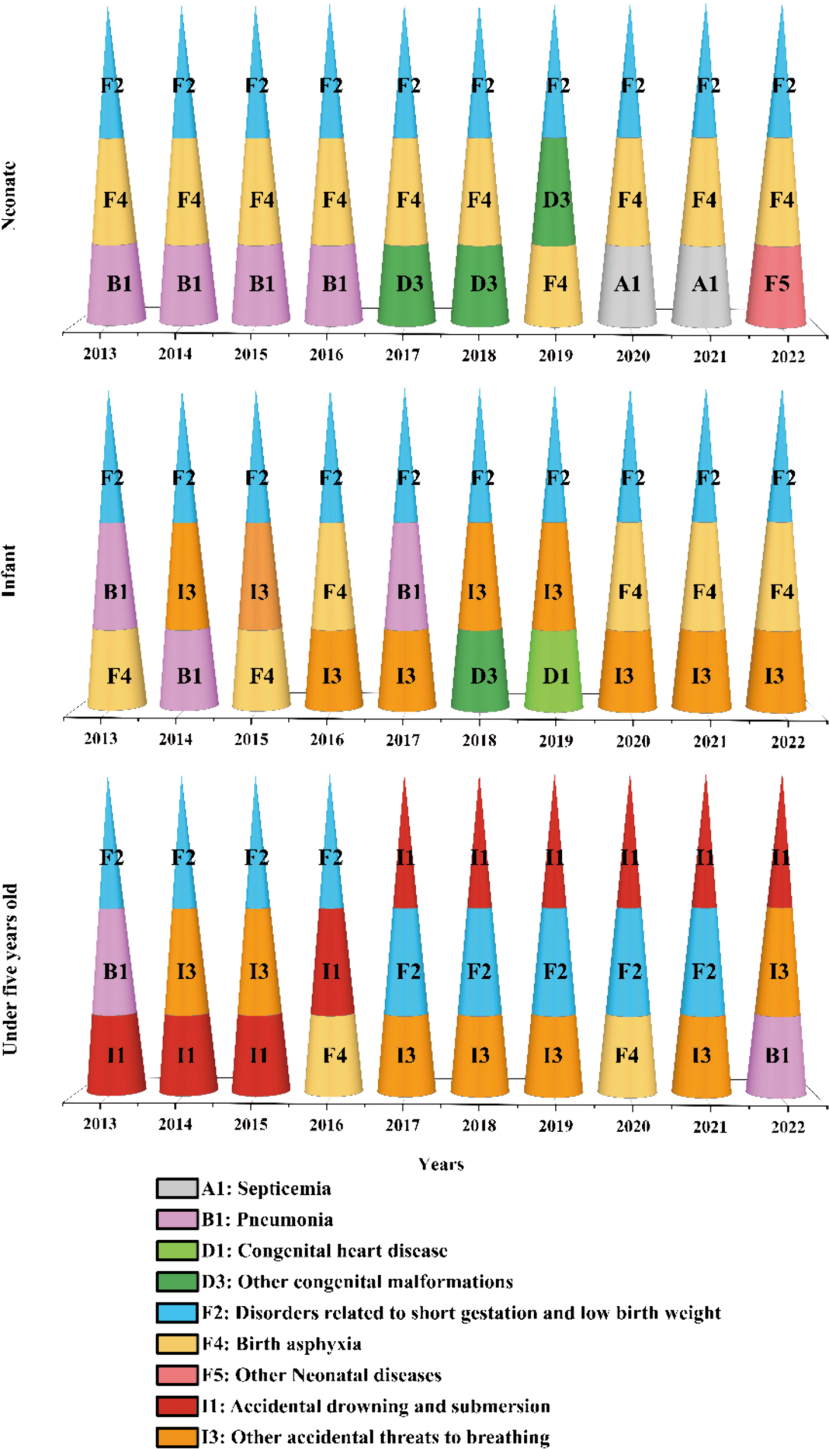
The primary causes of mortality among neonates, infants, and children under 5 years of age in Huangshi, 2013–2022

Figure 4 depicts the evolution of leading causes of mortality among neonates, infants, and children under 5 years old from 2013 to 2022. Over this decade, F2: Disorders related to short gestation and low birth weight, consistently ranked as the primary cause of neonatal and infant deaths.

For neonatal mortality, F4: Birth asphyxia, was the second most common cause, except for 2019 when D3: Other congenital malformations took its place. The third leading cause varied significantly, including B1: Pneumonia, D3, A1: Septicemia, F4, and F5: Other neonatal diseases (Fig. 4).

In infant mortality, F4: Birth asphyxia, I3: Other accidental threats to breathing, and B1: Pneumonia, were the second leading causes, while the third leading causes were more varied, encompassing I3, F4, B1, D1: Nutritional deficiencies, and D3: Other congenital malformations (Fig. 4).

For children under 5, disorders related to F2: Short gestation and low birth weight, was the leading cause of death from 2013 to 2016, shifting to I1: Accidental drowning and submersion, from 2017 to 2022. The second leading cause included F2 (2017 to 2021) and then a variety of factors such as I3: Other accidental threats to breathing, B1: Pneumonia, and I1. The third leading causes were I3, I1, F4: Birth asphyxia, and B1: Pneumonia (Fig. 4).

As children age, the leading causes of death diversify significantly, highlighting different risk factors for neonates, infants, and children under 5. This indicates a shift in mortality risks as children grow, with varied primary causes across different age groups.

## Discussion

During the 1980s, the Chinese government intensified its efforts to address maternal mortality and U5MR. Committed to achieving the Millennium Development Goals and Sustainable Development Goals set by the World Health Organization, China has seen remarkable progress in reducing U5MR since 2000 [14]. This achievement stems from comprehensive national, societal, individual, and economic initiatives.

In line with these national goals, Huangshi has made significant strides in lowering its U5MR, which plummeted from 21.38 per 1,000 live births in the early 2000s to an impressive 3.53 per 1,000 by 2022. This marked improvement reflects two decades of concerted efforts, with the infant mortality rate falling from 16.72 per 1,000 live births in 2002 to just 1.66 per 1,000 in 2022.

The global U5MR decreased from 71.2 to 38.1 per 1,000 live births between 2002 and 2021, marking a decline of almost 33.1 per 1,000 live births over the past two decades. In China, the U5MR dropped by 24.6 per 1,000 live births, from 31.5 to 6.9 per 1,000. In Huangshi, the U5MR decreased by 17 per 1,000 live births, from 21.4 to 4.4 per 1,000. Compared to global and national figures, Huangshi has demonstrated superior performance in reducing the U5MR.

Huangshi has significantly reduced the mortality rate of children under five years old, a success closely linked to the ongoing improvement of medical and healthcare services in the area. In 2002, there were only 113 healthcare facilities with 7,199 beds in Huangshi. By the end of 2022, the number of health facilities had expanded to 1,390, encompassing 50 hospitals, 38 community health service centers, 38 health centers, three maternal and child healthcare centers, three disease prevention and control centers, and staffed by 21,079 health professionals. Among these professionals, 7,275 were practicing and assistant physicians, while 10,065 were registered nurses. The total bed capacity in medical institutions increased to 19,384.

In the early 21st century, Huangshi experienced a high under-five mortality rate, primarily attributable to the following factors:

1. *Infectious Diseases*: Malaria, pneumonia, and diarrhea were prevalent across many parts of China, including Huangshi. These diseases posed significant risks to infants and young children, especially in economically disadvantaged regions.
2. *Malnutrition*: Certain regions and families faced malnutrition, which stunted children’s growth and weakened their immune systems. This vulnerability increased their disease risk and under-5 mortality.
3. *Healthcare Access*: Limited access to basic healthcare and sanitation in some areas impeded the provision of timely and effective medical care for sick children.
4. *Environmental Conditions*: Pollution and poor living conditions in some areas negatively affected children’s health, raising the risk of diseases and mortality.
5. *Socio-economic Challenges*: In low-income areas, families’ limited socio-economic resources hindered access to adequate childcare and medical services.
6. *Education and Awareness*: Lower levels of health literacy in some communities resulted in insufficient knowledge of preventive and treatment options for children’s diseases, complicating effective healthcare practices for parents.

A detailed investigation into mortality factors was conducted among a cohort of 1,929 children, categorizing them into 35 distinct subgroups. The primary causes of child mortality were identified as F2: Disorders related to short gestation and low birth weight, I1: Accidental drowning and submersion, and I3: Other accidental threats to breathing, all of which are largely preventable.

This analysis highlights the urgent need to prioritize child welfare and emphasizes the necessity for governmental policies focused on protecting children’s well-being. Furthermore, a detailed analysis of the leading causes of mortality within specific age groups under five years provided crucial insights for parents to address their concerns and improve childcare practices.

Over the last decade, the data showed a higher incidence of male fatalities compared to females, with a gender ratio of 1.38. The tendency of boys to engage in more active, adventurous, and unrestrained behavior may increase their risk of accidents, potentially leading to harm or injury. Such behavioral differences could account for the higher risk of injury and mortality among boys [15].

We sourced global and Chinese U5MR from 1990 to 2021, including sex-specific mortality rates, from the WHO. The data reveal that globally and within China, the mortality rate for boys under five exceeds that of girls, with the gender disparity widening from 1.05 to 1.08 in 1990 to 1.12–1.13 in 2021. Analyzing data from Huangshi, we hypothesize that as the mortality rate for children under five declines annually, the gender disparity may expand. The text discusses the higher susceptibility of boys to fatal accidents and urges parents to enhance child education and vigilance towards physical and mental health, aiming to lower the U5MR.

The primary causes of mortality among children under 5 years old vary significantly with age, reflecting distinct characteristics of each age group. Neonatal and infant deaths are mainly attributed to F2: Disorders related to short gestation and low birth weight. This underscores a critical link between gestational duration, birth weight, and child survival. In the broader category of children under 5, F2: Disorders related to short gestation and low birth weight, along with I1: Accidental drowning and submersion, stand out as the leading causes of death, pointing to a significant association with gestational length, birth weight, and accidents under parental supervision. As children age, the leading causes of death under the age of five evolve, necessitating robust governmental measures to safeguard children’s physical and mental health. It also demands that parents invest more time and effort in accompanying and caring for their children, ensuring a safer and healthier upbringing.

Additionally, several cities in China, including Huangshi and Xuzhou [16], have achieved remarkable reductions in the rates of newborn, infant, and under-five mortality. These achievements are largely due to China’s comprehensive health policies and interventions, which include increased focus and investment in child health, improved primary medical services, enhanced health standards, and widespread vaccination efforts. The key factors behind these successes include:

7. *Enhancement of Basic Medical Services*: China has committed to raising the quality of primary medical care, which includes building facilities and training healthcare personnel to ensure more children receive timely and effective medical assistance [17, 18].
8. *Vaccination Program*: China has launched a widespread childhood vaccination initiative, covering vaccines for infectious diseases such as measles, whooping cough, and polio. This extensive immunization effort has markedly reduced the incidence of numerous preventable diseases in children [19–21].
9. *Maternal Health:* Enhancing maternal health services and improving care quality during pregnancy and childbirth are crucial to lowering newborn and infant mortality rates [22, 23].
10. *Nutritional Improvement*: China has taken steps to improve children’s nutritional status, including supplementing nutrition for infants and young children, encouraging breastfeeding, and increasing the availability and safety of food [24, 25].
11. *Health Education*: Health education campaigns have been instrumental in raising parents’ and communities’ awareness about child health, promoting positive health behaviors, preventing diseases, and ultimately reducing mortality rates [26, 27].
12. *Sanitation and Hygiene*: Improving basic sanitation infrastructure, such as water supplies and toilets, plays a vital role in reducing child morbidity and mortality rates [28, 29].

The holistic approach to these initiatives has significantly enhanced children’s health and reduced child mortality, highlighting China’s unwavering dedication to improving its public health and medical systems.

Our study has certain limitations: (1) The scarcity of data on child mortality between 2002 and 2012 limited our capacity for deeper analysis during this timeframe. (2) Urbanization has blurred the lines between urban and rural areas, yet significant differences in under-five mortality rates (U5MR) remain between these environments. (3) In analyzing causes of death, the existence of unidentified causes may introduce the risk of inaccurate conclusions or misinterpretations.

## Conclusion

In the past two decades, Huangshi has achieved notable success in lowering the U5MR, with both its infant and neonatal mortality rates now falling below the national average.

Upon examining the main causes of mortality in children under five years of age, it is clear that significant efforts are needed to reduce the U5MR. This study focuses on analyzing trends in mortality rates among children under five and the leading causes of death in Huangshi from 2002 to 2022. The paper calls for the government to adopt more effective interventions to enhance the living conditions for children. Additionally, it encourages parents to increase their awareness of their children’s healthy development.

### Supplementary Information

Supplementary Material 1.

## Data Availability

The processed data was available in the paper, and raw data is freely serviced from first and corresponding author.
