# Supplementary material for: Retrospective analysis of mortality among children under 5 years of age in Huangshi over the period 2002–2022, China
Source: BMC Public Health. 2024 May 29;24:1431. doi: 10.1186/s12889-024-18955-3 (PMC11134869; doi:10.1186/s12889-024-18955-3)
Supplement: Supplementary file 1 — Supplementary Material 1. [file 12889_2024_18955_MOESM1_ESM.docx]

**Supplemental table 1. The Classification of the etiologies of child mortality.**

| Causal classification of child deaths | |
| --- | --- |
| A: Certain infectious and parasitic diseases | A1: Septicemia |
|  | A2: Other infectious and parasitic diseases |
|  | A3: Tuberculosis |
|  | A4: Dysentery |
|  | A5: Measles |
| B: Diseases of the respiratory system | B1: Pneumonia |
|  | B2: Other diseases of the respiratory system |
| C: Diseases of the digestive system | C1: Diarrhea |
|  | C2: Other diseases of the digestive system |
| D: Congenital malformations, deformations and chromosomal | D1: Congenital heart disease |
|  | D2: Down syndrome |
|  | D3: Other congenital malformations |
|  | D4: Neural tube defects |
| E: Diseases of the nervous system | E1: Meningitis |
|  | E2: Other disorders of the nervous system |
| F: Neonatal diseases | F1: Sclerema neonatorum |
|  | F2: Disorders related to short gestation and low birth weight |
|  | F3: Intracranial haemorrhage |
|  | F4: Birth asphyxia |
|  | F5: Other Neonatal diseases |
|  | F6: Tetanus neonatorum |
| G: Neoplasms | G1: Leukaemia |
|  | G2: Other neoplasms |
| H: Diseases of other systems | H1: Diseases of the circulatory system |
|  | H2: Endocrine, nutritional and metabolic diseases |
|  | H3: Diseases of the blood and blood-forming organs |
|  | H4: Diseases of the genitourinary system |
| I: Accidental injury and poisoning | I1: Accidental drowning and submersion |
|  | I2: Transport accidents |
|  | I3: Other accidental threats to breathing |
|  | I4: Accidental falls |
|  | I5: Other accidental injury |
|  | I6: Accidental poisoning by and exposure to noxious substances |
| J: Other definite disease | **——** |
| K: Ill-defined and unknown causes of death | **——** |

**Note:** There were no reported instances of children succumbing to dysentery, measles, tuberculosis, or tetanus neonatorum in Huangshi.

**Supplemental figure 1**

**
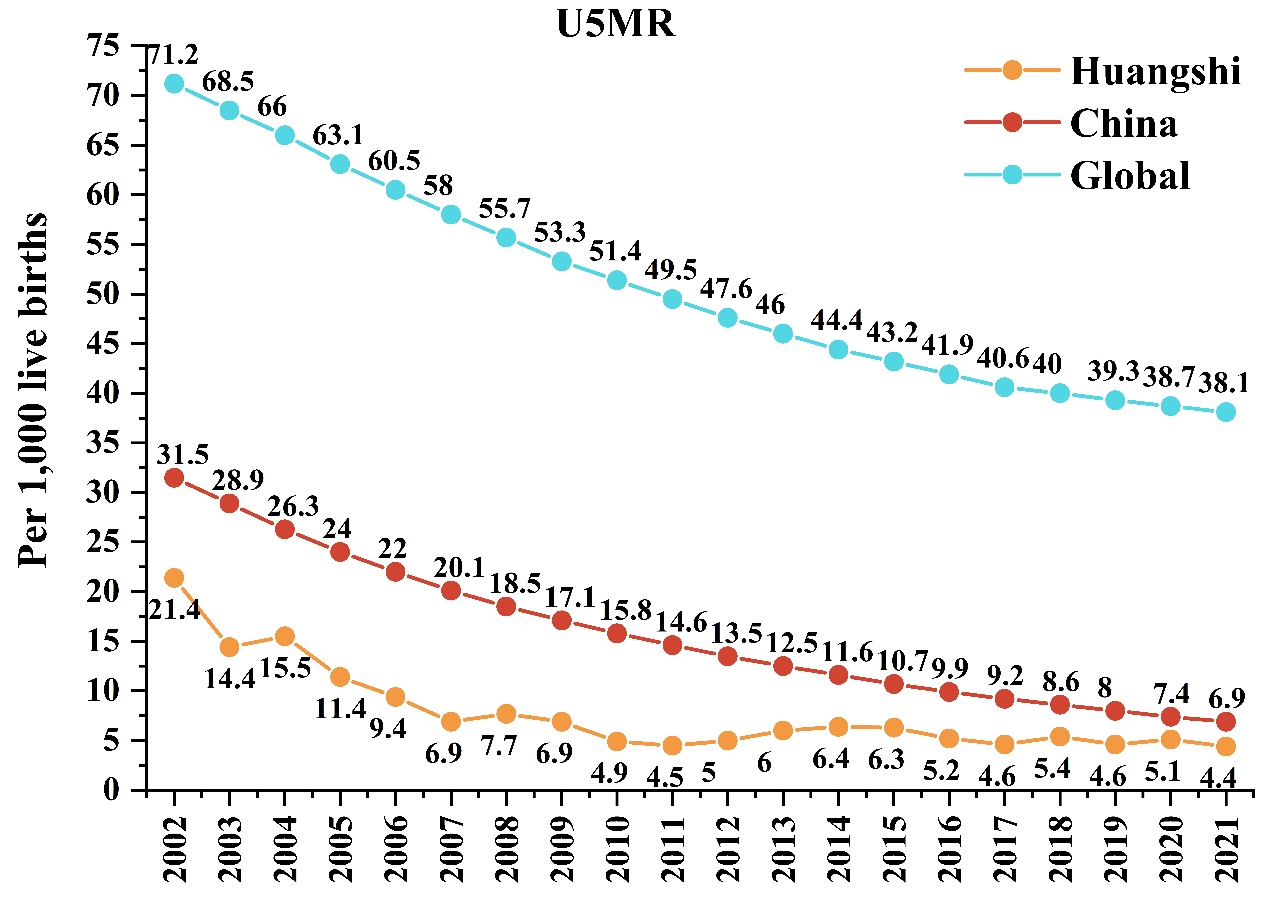
**

**Supplemental figure 1. Under-5 mortality rate of global, China, and Huangshi, from 2002 to 2021.**
